# Arctigenin Treatment Protects against Brain Damage through an Anti-Inflammatory and Anti-Apoptotic Mechanism after Needle Insertion

**DOI:** 10.3389/fphar.2016.00182

**Published:** 2016-06-22

**Authors:** Jie Song, Na Li, Yang Xia, Zhong Gao, Sa-feng Zou, Liang Kong, Ying-Jia Yao, Ya-Nan Jiao, Yu-Hui Yan, Shao-Heng Li, Zhen-Yu Tao, Guan Lian, Jing-Xian Yang, Ting-Guo Kang

**Affiliations:** ^1^School of Pharmacy, Liaoning University of Traditional Chinese MedicineDalian, China; ^2^Department of Engineering, St. Cross College, University of OxfordOxford, UK; ^3^Department of Interventional Therapy, Department of Rehabilitation, Dalian Municipal Central HospitalDalian, China

**Keywords:** arctigenin, stab wound injury, inflammation, apoptosis, convection enhanced delivery, traumatic brain injury

## Abstract

Convection enhanced delivery (CED) infuses drugs directly into brain tissue. Needle insertion is required and results in a stab wound injury (SWI). Subsequent secondary injury involves the release of inflammatory and apoptotic cytokines, which have dramatic consequences on the integrity of damaged tissue, leading to the evolution of a pericontusional-damaged area minutes to days after in the initial injury. The present study investigated the capacity for arctigenin (ARC) to prevent secondary brain injury and the determination of the underlying mechanism of action in a mouse model of SWI that mimics the process of CED. After CED, mice received a gavage of ARC from 30 min to 14 days. Neurological severity scores (NSS) and wound closure degree were assessed after the injury. Histological analysis and immunocytochemistry were used to evaluated the extent of brain damage and neuroinflammation. Terminal deoxynucleotidyl transferase dUTP nick end labeling (TUNEL) was used to detect universal apoptosis. Enzyme-linked immunosorbent assays (ELISA) was used to test the inflammatory cytokines (tumor necrosis factor (TNF)-α, interleukin (IL)-6 and IL-10) and lactate dehydrogenase (LDH) content. Gene levels of inflammation (TNF-α, IL-6, and IL-10) and apoptosis (Caspase-3, Bax and Bcl-2) were detected by reverse transcription-polymerase chain reaction (RT-PCR). Using these, we analyzed ARC’s efficacy and mechanism of action. Results: ARC treatment improved neurological function by reducing brain water content and hematoma and accelerating wound closure relative to untreated mice. ARC treatment reduced the levels of TNF-α and IL-6 and the number of allograft inflammatory factor (IBA)- and myeloperoxidase (MPO)-positive cells and increased the levels of IL-10. ARC-treated mice had fewer TUNEL+ apoptotic neurons and activated caspase-3-positive neurons surrounding the lesion than controls, indicating increased neuronal survival. Conclusions: ARC treatment confers neuroprotection of brain tissue through anti-inflammatory and anti-apoptotic effects in a mouse model of SWI. These results suggest a new strategy for promoting neuronal survival and function after CED to improve long-term patient outcome.

## Introduction

Traumatic brain injury (TBI) is acquired from an external force, which can inflict devastating effects to the brain tissue, vasculature and neighboring neuronal cells (Logsdon et al., 2015). CED infuses drugs directly into brain tissue (Casanova et al., 2014a), which can result in stab wound injury (SWI), which is a type of TBI.

The success of neurological disorder treatment depends on effective techniques that deliver drugs to the central nervous system (CNS). However, the blood–brain barrier (BBB) prevents passive passage of the majority of large molecules from the bloodstream into the extracellular space, so systemic delivery can be problematic (Abbott and Romero, 1996; Nicholson, 2001). CED is a technique that delivers therapeutic drugs directly to the CNS, bypassing the BBB (Bobo et al., 1994). In CED, a needle is inserted directly into the brain, and drug infusate is delivered at controlled flow rates into the nidus. With convection as the dominant transport mechanism, this technique can improve local delivery and absorption by providing larger distribution volumes than systemic delivery methods (Bobo et al., 1994; Casanova et al., 2014a). While CED provides a new and effective method for the treatment of nervous system diseases, at the same time, it will cause the injury in the treatment process of insertion.

Convection enhanced delivery requires the surgical insertion of a needle into brain tissue, and this results in SWI. The primary damage of SWI is tissue tear, cell breakage and hemorrhage. However, subsequent secondary injury is the main effect on wound recovery after SWI, includes brain edema, hematoma, and inflammatory and apoptotic responses, etc (Logsdon et al., 2015). Although we have an improved understanding of the pathophysiology that occurs in mechanical brain injury, clinical neuroprotection trials that attempt to pharmacologically prevent cellular death after mechanical brain injury have failed to show consistent improvement in outcome for these patients. The lack of effective therapy to repair injured brain tissue has motivated researchers to focus on stem cells as a potential avenue for regeneration, though much evidence of functional recovery was provided by the use of exogenous stem cells in brain injury in rodent models. However, there are still many questions to be answered, including ethical and theoretical issues, the appropriate source of stem cells, and immune rejection. Due to the lack of effective therapies, the main clinical treatment is to administer nerve nutrition and dehydration drugs, but the effect is not ideal for injury and nervous system recovery. Inflammation and apoptosis is a pathological hallmark of secondary injury of SWI. The focus of modern medical treatment is to control and reduce secondary damage. Seeds of Arctium lappa have been used as a diuretic, anti-inflammatory and detoxifying agent in Chinese traditional medicine. ARC, a bioactive phenylpropanoid dibenzylbutyrolactone component lignin isolated from Arctium lappa, has anti-inflammatory, and anti-apoptotic activities (Lee et al., 2010; Tsai et al., 2011; Gu et al., 2012; Zhang N. et al., 2013).

The release of inflammatory cytokines in cells after injury is a normal immune response, but the overexpression of some pro-inflammatory cytokines, such as IL-6 and TNF-a, are detrimental to wound recovery (Zhai et al., 2015). A previous study by Zhang et al. (2015) reports that ARC decreased lipopolysaccharide (LPS)-induced acute lung inflammation, infiltration of inflammatory cells into bronchoalveolar lavage fluid, and production of pro-inflammatory cytokines. In other reports, ARC had significantly decreased not only carrageenan-induced paw edema but also MPO and eosinophil (EPO) activities in arachidonic acid-induced edematous tissues (Kang et al., 2008).

However, some anti-inflammatory cytokines, such as IL-10, are beneficial to injury recovery (Zhai et al., 2015). Thus, a drug that would increase favorable cytokines and reduce adverse cytokines is best for disease recovery. Hyam et al. (2013) observed that ARC not only peritoneal macrophages but also increased LPS-reduced IL-10 and a cluster of differentiation (CD) 204 expression.

Many plant products have neuroprotective effects such as osthole and ashwagandha. Osthole confers neuroprotection against cortical SWI and attenuates secondary brain injury (Xia et al., 2012). Ashwagandha and its constituent withanolide A confers neuroprotection against β-amyloid and HIV-1Ba-L (clade B) induced neuro-pathogenesis (Kurapati et al., 2013, 2014). These drugs are derived from traditional medicines which have advantages on relatively minor toxic side effects and long-term usage; it would be helpful to treat diseases of the nervous system. ARC has been shown to act on scopolamine-induced memory deficit mice and to provide a neuroprotective effect on cultured cortical neurons. In our previous study, we investigated the neuroprotective effect of ARC on H89-induced cell damage and its potential mechanisms in mouse cortical neurons and human SH-SY5Y neuroblastoma cells. We found that ARC prevented cell viability loss and reduced intracellular beta amyloid (Aβ) production induced by H89 in neurons and human SH-SY5Y cells and also inhibited presenilin 1 (PS1) protein level in neurons. Our results showed that ARC confers neuroprotective effects via upregulation of phospho-cAMP response element binding protein (P-CREB) in mouse primary neurons and human SH-SY5Y neuroblastoma cells. The reduction of inflammation can contribute to nervous system recovery, improving the neurological dysfunction caused by the injury. A previous study has shown that ARC treatment significantly reduced cerebral infarction and improved neurological outcome through suppressing the activation of microglia and decreasing the expression of IL-1β and TNF-α (Fan et al., 2012).

The release of apoptotic cytokines is a major factor affecting wound and neurological function recovery (Zhai et al., 2015). Inflammation and apoptosis are closely related, and inflammation can cause apoptosis (Fernandes-Alnemri et al., 2009). Therefore, effective drugs can be both anti-inflammatory and anti-apoptotic. In experimental Japanese encephalitis (JE) treatment, ARC provided complete protection against disease. ARC’s neuroprotective effect was associated with a marked decreases in microgliosis and proinflammatory cytokines, active caspase-3 activity and neuronal death. Furthermore, treatment with ARC also improves the behavioral outcome following JE (Swarup et al., 2008).

Based on the above studies, we assume that ARC treatment can protect against brain damage via anti-inflammatory and anti-apoptotic effects in a mouse model of CED-induced brain injury. We observed neurological function, the BBB, inflammation and apoptosis following a 2-week treatment with ARC. The results suggest a new strategy for restoring neuronal function and improving long-term patient outcomes after CED.

## Materials and Methods

### Preparation of ARC

**FIGURE 1:**
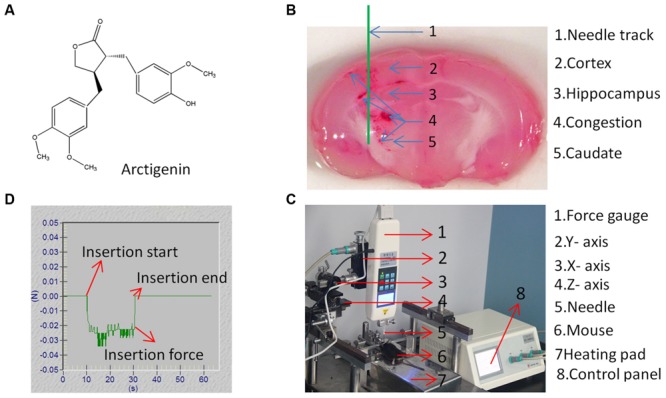
**Structure of arctigenin (ARC) and CED system. (A)** The chemical structure of ARC. **(B)** Coronal section of the mouse brain showing a schematic of the needle track (in green). **(C)** Diagram of mouse model showing the CED system placement and site of stab in SWI brain injury. **(D)** Typical needle insertion force vs. time for brain tissue. The insertion speed was 0.2 mm/s, and the insertion force was measured by digital force gage.

Arctigenin (catalog no. 140524, purity > 98% (**Figure 1A**) was purchased from the Chengdu Puei De Biotech Co., Ltd (SIchuan, China), dissolved in sodium carboxyl methyl cellulose (5% CMC) (Kong et al., 2015), and stored at 4°C.

### Animals

Adult (25–30 g) C57BL/6J mice aged 3–4 months were housed under a 12:12-h light/dark cycles with free access to food and water. Animal procedures conformed to guidelines set by Liaoning University of Traditional Chinese Medicine Institutional Animal Care and Use Committee, which are in accordance with those set by the National Institutes of Health (Bethesda, MD, USA).

### Model

A mouse model of SWI, as previously described in (Xia et al., 2012; Hu et al., 2013; Gao et al., 2014) with slight modifications, was created to mimic the CED procedure. Briefly, anesthesia was initiated with chloral hydrate (4%, 0.1 ml/10 g) delivered at 1.0 ml/min. The head was shaved and disinfected with iodine/alcohol. Animals were fixed on an Autostereotaxic Frame (PTW-1, Chengdu Instrument Factory, China). Body temperature was maintained (37°C) with a heating pad during the entire procedure. The skull was exposed by a mid-sagittal incision that began between the eyes and extended caudally to the level of the ears to expose the bregma and lambda. A hole of 2-mm diameter was drilled by hand into the skull above the caudate putamen (CPu) over the left cerebral hemisphere. Dura mater was carefully taken off, and any residual blood was cleaned with phosphate–buffered saline (1 × PBS). A 20-gage, 1.5-mm diameter needle (Hamilton Company, Reno, NV, USA), with a rigid core was inserted 2.5 mm lateral to the midline, 2.5 mm posterior to the lambdoidal suture, and at a depth of 4 mm from the surface of the brain (**Figure 1B**). The exposed surface of the brain was kept wet with PBS during the course of the experiment. The rigid core was then withdrawn, and the needle was left *in situ* for 5 min before removal. The skin was then sutured, and mice were allowed to recover in their cages.

### Needle Insertion and CED System

According to studies by Casanova et al. (2014a,b), a stainless steel needle with a blunt tip was used in this study because it has been used in clinical trials. To minimize tissue entering the needle tip, the inner lumen of the needle was filled with cyanoacrylate glue. To ensure the consistency of the experiment, a linear stage (**Figure 1D**) was used to control needle insertion. The needle was mounted on the *Y*-axis of the linear stage, and mice were fixed on the heating pad with the metal frame to position the mice under the needle. The *X*-axis and *Z*-axis of the linear stage were adjusted such that drilled holes in the rat skull were aligned with the needle tip. The *Y*-axis of the linear stage was adjusted such that the needle tip just reached the brain, and subsequently, needles were inserted to a depth of 4 mm into the brain. To reduce deviation, all processes of needle insertion were controlled by a numerical control panel. The dura mater is considered to be a tough membrane covering that would add excessive surface deformation during needle insertion. Thus, the dura mater was carefully removed before needle insertion. Studies by Casanova et al. (2014a,b) indicated that different speeds of needle insertion can produce different pressures, which can induce different extents of damage. To minimize the effects of speed and pressure changes during insertion, a constant speed (0.2 mm/s) was used that produced a stable pressure (0.029 ± 0.005 N) (**Figure 1C**). The insertion force was measured by digital force gage (HF-2, Wenzhou tripod instrument manufacturing CO., LTD, China). The digital force gage monitored the process of insertion in real-time. To reduce injury induced by infusion pressure, a low drug infusion speed was used (2 μL/min). We assume that the dosage of infusion was 10 μL, so the infusion process takes approximately 5 min. Therefore, to mimic the drug delivery process, the needle must stay in mice for 5 min after needle insertion.

### Drug Use

Mice were randomly divided into five groups (*n* = 6 each): mice in groups 1–3 (SWI+ARC) were administered ARC intragastrically (ig) 30 min after surgery at 20, 40, and 80 mg/kg dissolved in 5% CMC in PBS followed by once daily intragastrical (ig) administration (200 μl) for the next 14 days; mice in the SWI control group were given 5% CMC in PBS by intragastrical (ig) administration (200 μl) each day for 14 days; and naïve C57BL/6J mice were used as controls. Mice were sacrificed between 3 and 21 days post injury (dpi) for analyses.

### Assessment of Neurological Function

Neurological function was assessed with a modified NSS at 24 h and 3, 7, 14, and 21 dpi, as previously described (Lu et al., 2003; Hirjak et al., 2013; Liu et al., 2014). The NSS evaluation consisted of motor, sensory, reflex, and balance tests, with results measured on a scale of 0–18 (0 = normal, 1–6 = mild injury, 7–12 = mean-moderate injury; 13–18 = severe injury, and 18 = maximal deficit). The test was administered by blinded, trained investigators, and mice were familiarized with the testing environment before being subjected to brain injury.

### Measurement of Brain Water Content

Brain water content was measured 3 days post-SWI. Following decapitation, brains were harvested, and the cerebellum and olfactory bulb was removed. Subsequently, surface liquid was dried with filter paper. The brain wet weight was obtained on a pre-weighed metal box after drying in an electric oven at 105°C for 24 h, and the percent water was calculated as (wet weight – dry weight)/(wet weight) (Lee et al., 2008; Taya et al., 2010; Xia et al., 2012).

### Determination of Albumin Leakage and Brain Hematoma

Blood–brain barrier permeability was determined by measuring Evans blue (EB) extravasation (Başkaya et al., 1997; Shi et al., 2015). Four percent EB (4 mL/kg; Sigma–Aldrich) was injected into the caudal vein at 3 dpi. One hour later, animals were transcardially perfused with cold saline to remove intravascular EB. The brains were removed carefully and visualized on a OLYMPUS SZX9 microscope (Tokyo, Japan) with a digital camera.

To quantify EB extravasation, each brain was carefully weighed and soaked in methanamide for 48 h at 37.0°C, then subsequently centrifuged for 30 min at 20,000 *g*. The absorption of the supernatant was measured at 632 nm with a spectrophotometer (Bio-Rad, Hercules, CA, USA). Tissue EB concentration was quantified using a standard curve and expressed as μg/g of brain tissue.

Brain hematoma volume was determined by ImageJ calculation of the area of congestion at 3 dpi. Mice were anesthetized with chloral hydrate (4%, 0.1 ml/10 g) then were transcardially perfused with 1% PBS to remove intravascular blood. The brains were removed carefully and visualized on a OLYMPUS SZX9 microscope (Tokyo, Japan) with a digital camera.

### Measurement of Lesion Size

At 3, 7, 14, and 21 dpi, mice were anesthetized and transcardially perfused with 4% paraformaldehyde in cold phosphate buffer. Brains were immediately harvested and visualized on a OLYMPUS SZX9 microscope (Tokyo, Japan) with a digital camera. The size of the wound lesion was measured in each brain by tracing a line along the edge of the tissue lining the lesion with ImageJ software (National Institutes of Health) (d’Avila et al., 2012). Measurements were taken from 6 brains at each time point.

### Slice Preparation

Mice were anesthetized with 4% chloral hydrate (0.1 ml/10 g, i.p.), and then transcardial perfusion with 1% PBS was performed. Subsequently, 4% paraformaldehyde was perfused to fix tissue. The brains were harvested and cryo-protected in PBS containing 30% sucrose until brains sank to the bottom. Then, the brains were equilibrated to optimum cutting temperature compound (OTC) and placed in the freezer. The brains were sectioned into 5–10 mm thick sections with a cryostat (CM1900, Leica). The sections were located in a direction parallel to the needle penetration line at 150 μm intervals to cover the entire lesion site. The sections were mounted on glass slides for staining and visualized on a OLYMPUS SZX9 and BX51 microscope (Tokyo, Japan) with a digital camera.

### Nissl Staining

Seven days after SWI, the mouse brains (six per group) were harvested and made into 5 μm slices for Nissl staining (Zhao et al., 2014; Zhai et al., 2015). The sections were fixed in absolute ethyl alcohol and then processed through different baths in the following order: 100% ethanol (1 min), 95% ethanol (1 min), 70% ethanol (1 min), double distilled water (1 min, three times), cresyl violet (56°C, 1 h), double distilled water (1 min, three times), neutral differentiation solution (2 min), 100% ethanol (1 min), xylene (1 min); the samples were then mounted with neutral balata and covered with a coverslip. The Nissl staining sections were visualized on a OLYMPUS SZX9 and BX51 microscope (Tokyo, Japan) with a digital camera.

### Hematoxylin and Eosin (H&E) Staining

At 3 dpi, the mouse brains (six per group) were harvested and made into 10 μm slices for H&E staining (Bitto et al., 2012; Casanova et al., 2014a,b). The sections were dried for 30 min at room temperature and then processed through different baths in the following order: 70% ethanol (5 s), 80% ethanol (5 s), 95% ethanol (5 s), distilled water (5 s, three times), hematoxylin (5 s, three times), alcohol lamp heating (ten times), distilled water (1 min, three times), 1% alcohol differentiation solution (3 s, five times), distilled water (1 min, three times), 5% ammonia (3 s, eight times), distilled water (1 min, three times), eosin (10 times), distilled water (1 min, three times), 70% ethanol (5 s), 80% ethanol (5 s), 95% ethanol (5 s), alcohol lamp heating; the samples were then mounted with neutral balata and covered with a coverslip. The H&E staining sections were visualized on a OLYMPUS SZX9 microscope (Tokyo, Japan) with a digital camera.

### Immunofluorescent Staining

After SWI, the mice (six per group) were sacrificed at 3 days for inflammation examination (Zhang R. et al., 2013) and 7 days for apoptosis examination (Liu et al., 2014). Brains were harvested and made into 5 μm slices for immunofluorescent staining. The sections were processed through different baths in the following order: 4% paraformaldehyde (30 min), 1% PBS (5 min, three times), 1% Triton X-100 (30 min), 1% PBS (5 min, three times), 5% bull serum albumin (BSA) (30 min), 1% PBS (5 min, three times), primary antibody (Rabbit Anti-GFAP antibody, Rabbit Anti-AIF1/Iba1 antibody, Rabbit Anti-MPO antibody, Rabbit Anti-NF-M antibody, Rabbit Anti-Caspase-3 antibody) (1:150, Abcam, Cambridge, MA, USA), incubation (4°C, 12 h), 1% PBS (5 min, three times), secondary antibody (Donkey Anti-rabbit IgG/Cy3 antibody, Donkey Anti-rabbit IgG/FITC antibody) (1:200, Jackson ImmunoResearch Lab, West Grove, PA, USA), incubation (protection from light, 1 h), 1%PBS (5 min, three times), 4′,6-diamidino-2-phenylindole (DAPI) (protection from light, 15 min), 1% PBS (5 min, three times); the samples were then mounted with antifade mounting medium and covered with a coverslip. The immunofluorescent staining sections were visualized on a OLYMPUS SZX9 microscope (Tokyo, Japan) with a digital camera.

### TUNEL Staining

TUNEL Universal Apoptosis Detection Kit (Roche, Chicago, IL, USA) was used for the fast detection of fragmented DNA in the nucleus by red fluorescence probe labels during apoptosis. Brain sections from mice at 7 dpi were analyzed for apoptotic cells according to the manufacturer’s instructions. The number of TUNEL-positive cells in each section in areas surrounding the lesion were counted in six sections per mouse and six mice per group using ImageJ software.

### Analysis of Cytokine Levels by ELISA

Brains were collected at 3 dpi, and the tissue around the cavity (diameter: 5 mm) weighing 100 mg was taken. Tissue homogenates were obtained in 900 μL 1% PBS, and the supernatant was stored at -80°C. LDH (novus biologicals, Littleton, USA, Cat. No. KA0878), TNF-α (Cat. No. MTA00B), IL-6 (Cat. No. M6000B), and IL-10 (Cat. No.M1000B) levels in the samples were measured using ELISA kits (R&D Systems, Minneapolis, MN, USA) following the manufacturer’s instructions (Xia et al., 2012).

### Reverse Transcription (RT)-PCR

Total RNA was extracted from brain tissue at 3 and 7 dpi with TRIzol reagent and reverse transcribed to cDNA using a RevertAid First Strand cDNA Synthesis kit (Thermo Scientific, Vilnius, Lithuania) (Yang et al., 2008). The PCR reaction (35 cycles) was performed using a DreamTaq Green PCR Master Mix Kit (Thermo Scientific). Quantitative RT-PCR was performed using the following forward and reverse primer sets designed using Premier Biosoft 5 (Palo Alto, CA, USA): TNF-α, 5′-ATA AGA GCA AGG CAG TGG AG-3′ and 5′-TCC AGC AGA CTC AAT ACA CA-3′;IL-6, 5′-AGC CAG AGT CCT TCA GAG AG-3′ and 5′-TCC TTA GCC ACT CCT TCT GT-3′; IL-10, 5′-TTC TCA TTC CTG CTT GTG GC-3′ and 5′-ATC TGA GTG TGA GGG TCT GG-3′; Bax, 5′-CTG ACA TGT TTT CTG ACG GC-3′ and 5′-TCA GCC CAT CTT CTT CCA GA-3′; Bcl-2, 5′-CGC TGG GAG AAC AGG GTA-3′ and 5′-GGG CTG GGA GGA GAA GAT-3′; Caspase-3, 5′-AGA TAC CGG TGG AGG CTG ACT-3′ and 5′-TCT TTC GTG AGC ATG GAC ACA-3′; and β-actin (control), 5′-GGG AAA TCG TGC GTG ACA T-3′ and 5′-TCA GGA GGA GCA ATG ATC TTG-3′. Products were resolved by 1.5% agarose gel electrophoresis with ethidium bromide staining. The mRNA levels of TNF-α, IL-6 and IL-10 were detected at 3 dpi, and those of Bax, Bcl-2, and caspase-3 were detected at 7 dpi. Quantitative analysis was performed using a Tanon 4100 Gel Imaging System (Tanon Science & Technology Co., Shanghai, China).

### Measuring Cell Fluorescence Using ImageJ

The fluorescent intensity was quantified by ImageJ, using the follow steps: Select the cell of interest using any of the drawing/selection tools (i.e., rectangle, circle, polygon, or freeform); From the Analyze menu select “set measurements”. Make sure that the AREA, INTEGRATED DENSITY, and MEAN GRAY VALUE have been selected; Select “Measure” from the analyze menu. A popup box will appear with a stack of values for that first cell; Select a region next to the cell that has no fluorescent, this will be located in the background; Repeat this step for the other cells in the field of view that needs to be measure; Once finished, select all the data in the Results window, copy and paste into a new excel worksheet; Use this formula to calculate the corrected total cell fluorescence (CTCF). More details of the operation can be viewed in the ImageJ’s instructions on Measuring Cell Fluorescence.

### Statistical Analysis

The data were analyzed using SPSS version 13.0 (SPSS, Chicago, IL, USA) and are presented as the mean ± SD. Differences between groups were assessed by one-way analysis of variance, and *post hoc* multiple comparisons were performed with the Student-Newman–Keuls test. *P* < 0.05 was considered statistically significant.

## Results

In the present study, we use naïve (uninjured) mice as controls rather than those subjected to craniotomy. In a previous study, minor injury craniotomy was shown to induce acute inflammatory response (Bayir et al., 2003; Cole et al., 2011). After craniotomy, mice had similar numbers of glial fibrillary acidic protein (GFAP)-positive astrocytes compared to experienced moderate cortical impact injury mice (Susarla et al., 2014).

### ARC Treatment Improves Neurological Function after SWI

**FIGURE 2:**
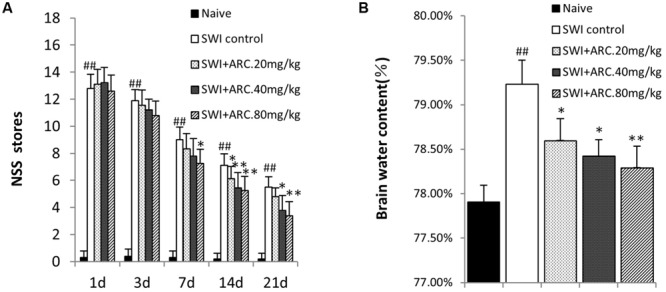
**Modified neurologic severity score and brain water content by ARC treatment. (A)** Neurological function was analyzed by NSS at 1, 3, 7, 14, and 21 days post injury (dpi). Treatment with ARC significantly lowered NSS from 3 to 21 dpi compared to untreated control. **(B)** Brain water content of injured hemispheres was measured at 3 dpi. The untreated SWI group had a significantly higher brain water content than naive control. ARC treatment significantly reduced brain water contents in a dose-dependent manner. *n* = 6 per group. The data are presented as the mean ± SD. ^##^*P* < 0.01 vs. naive control; ^∗^*P* < 0.05, ^∗∗^*P* < 0.01, vs. SWI control.

To determine whether ARC has neuroprotective effects after SWI, the curative effect of different concentrations of ARC (20, 40, or 80 mg/kg/day) were evaluated using an NSS system. NSS was evaluated from 1 to 21 dpi. As shown in **Figure 2A**, ARC treatment improves neurological function from 3 to 21 days compared to naïve control mice. ARC treatment decreased the NSS in a dose-dependent manner, and the administration of a high dose (80 mg/kg) decreased the NSS the most significantly compared to the middle and low dose. At 3 dpi, we observed that ARC showed a certain treatment effect of decreasing the NSS in various concentrations, but no significant difference was found between ARC-treated and SWI control group. Regarding 7 dpi, the treatment effect of ARC increased remarkable and had significant difference (7.25 ± 1.04 vs. 9.00 ± 0.93; *P* < 0.05 vs. SWI control at 7 dpi) between the highest dose group and that of SWI control. The treatment effect increased until 21 dpi in various concentrations (3.38 ± 1.06 vs. 5.50 ± 0.75; *P* < 0.01 vs. SWI control at 21 dpi).

### ARC Treatment Decreases Brain Edema Caused by Injury

Brain edema is caused by damage of the BBB after TBI, and brain water content increase is a marker for brain damage (Zhang R. et al., 2013). A second opening of the BBB will occur due to the inflammation response at 3 dpi (Başkaya et al., 1997). In the present study, different concentrations of ARC were tested to determine whether treatment can lower the brain water content by reducing the inflammatory response 3 days after injury. As shown in **Figure 2B**, the highest brain water content was in the SWI compared to the naïve control and administration group (79.23% ± 0.27% vs. 77.91% ± 0.19%); ARC treatment decreased the brain water content in a dose-dependent manner, and the most significant decrease was observed with 80 mg/kg ARC (78.29% ± 0.24%; *P* < 0.01 vs. SWI control).

### ARC Treatment Accelerates Wound Healing after SWI

**FIGURE 3:**
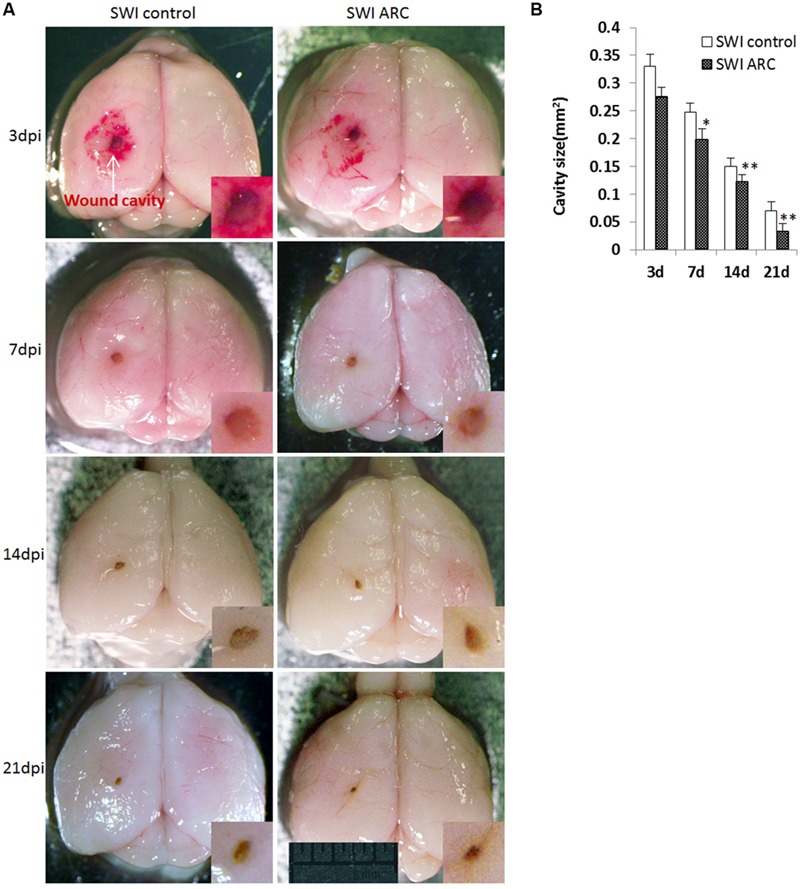
**Decreased wound cavity size in mice treated with ARC. (A)** Representative images of mouse brains at 3, 7, 14, and 21 dpi. **(B)** The graph shows the areas of wound cavities measured as described in the Section “Materials and Methods”. The cavity areas in ARC-treated mice from 3 to 21 dpi were significantly smaller than those in the untreated control. The wound cavity is shown at higher magnifications in the insets. Measurements were taken from 6 brains and 6 mice per time point. The data are presented as the mean ± SD. ^∗^*P* < 0.05, ^∗∗^*P* < 0.01, vs. SWI control. Scale bar: 5 mm.

The most direct way to analyze wound healing is to observe the wound size in the brain cavity caused by needle insertion. The size of the brain cavity was measured at four different time points (3, 7, 14, and 21 dpi) after injury. As shown in **Figure 3A**, the size of the brain cavity gradually becomes smaller with the passage of time, but the size of the ARC treatment group is smaller than SWI control mice at each time point. Cavity size was smallest at 21 dpi in ARC-treated mice, and thus, ARC treatment accelerates wound healing after SWI. By 21 dpi, the cavity was smallest in 3/6 ARC-treated mice vs. 5/6 untreated controls (0.033 ± 0.0041 mm^2^ vs. 0.070 ± 0.0054 mm^2^; *P* < 0.05) (**Figures 3A,B**).

### ARC Treatment Decreases Albumin Leakage and Brain Hematoma Caused by Injury

**FIGURE 4:**
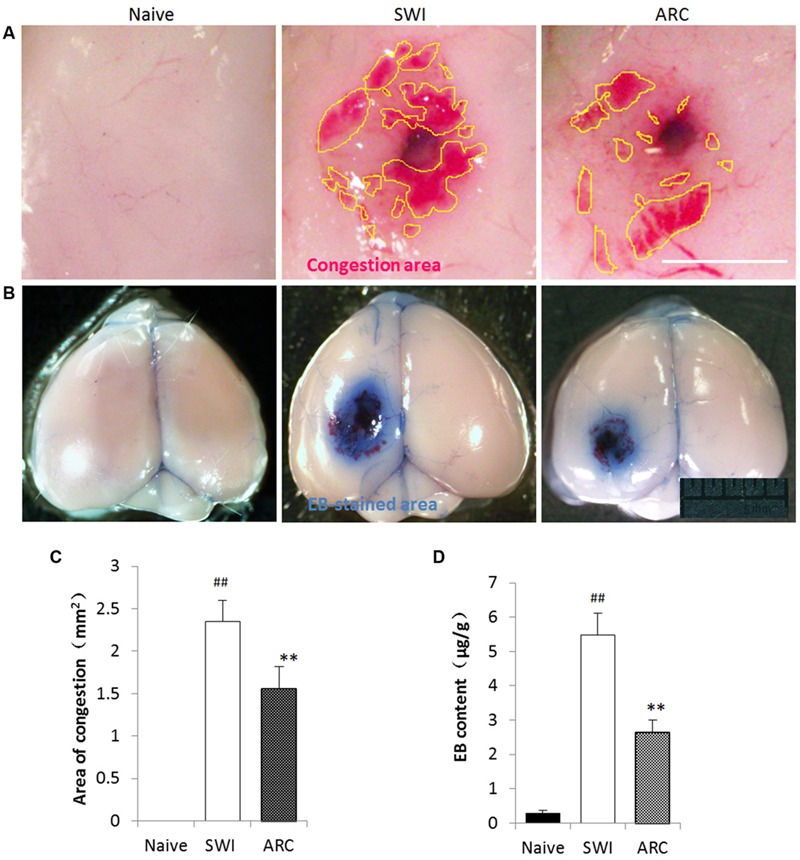
**Arctigenin treatment preserves BBB integrity and blood exudation after SWI. (A)** Representative images of the congestion area of the brain at 3 dpi after SWI. Yellow lines delimit the congestion area. The area of congestion was reduced in the ARC- treated SWI brain compared to the untreated brain. **(B)** Representative images of EB-stained brain at 3 dpi after SWI. The EB-stained areas were reduced in ARC-treated SWI brain compared to the untreated brain. **(C)** Quantitative results of the congestion area (*n* = 6/group) calculated by Image J software. **(D)** Quantitative results of EB extravasation in each brain (*n* = 6/group), expressed as EB content (μg/g) calculated from a standard curve. The data are presented as the mean ± SD. ^##^*P* < 0.05 vs. naive control; ^∗∗^*P* < 0.01, vs. SWI control. Scale bar: 0.5 mm in **(A)**, 5 mm in **(B)**.

Traumatic brain injury can inflict devastating effects to the brain vasculature and neighboring neuronal cells. Vasculature disruption and increased BBB permeability are primary effects that can lead to a host of secondary injury cascades (Logsdon et al., 2015). Primary damage of the BBB leads to brain hemorrhage and Albumin Leakage, which occur immediately after TBI, and a second opening of the BBB will occur due to the inflammation response at 3 dpi (Başkaya et al., 1997). In this study, EB leakage was tested to determine whether ARC treatment preserves BBB integrity via anti-inflammatory effects after SWI. As shown in **Figures 4B,D**, the volume of EB extravasation was significantly decreased in ARC-treated mice compared to SWI control mice (EB: 2.64 ± 0.36 μg/g vs. 5.47 ± 0.64 μg/g; *P* < 0.05). If ARC treatment protects BBB integrity, the volume of brain hemorrhage should also reduce. Consistent with this, brain hematomas were detected, and the result in **Figures 4A,C** shows that the volume of brain hematoma was significantly decreased in ARC-treated compared to SWI control mice (1.56 ± 0.26 mm^2^ vs. 2.35 ± 0.25 mm^2^; *P* < 0.05).

### ARC Treatment Suppresses the Inflammatory Response Caused by Injury

**FIGURE 5:**
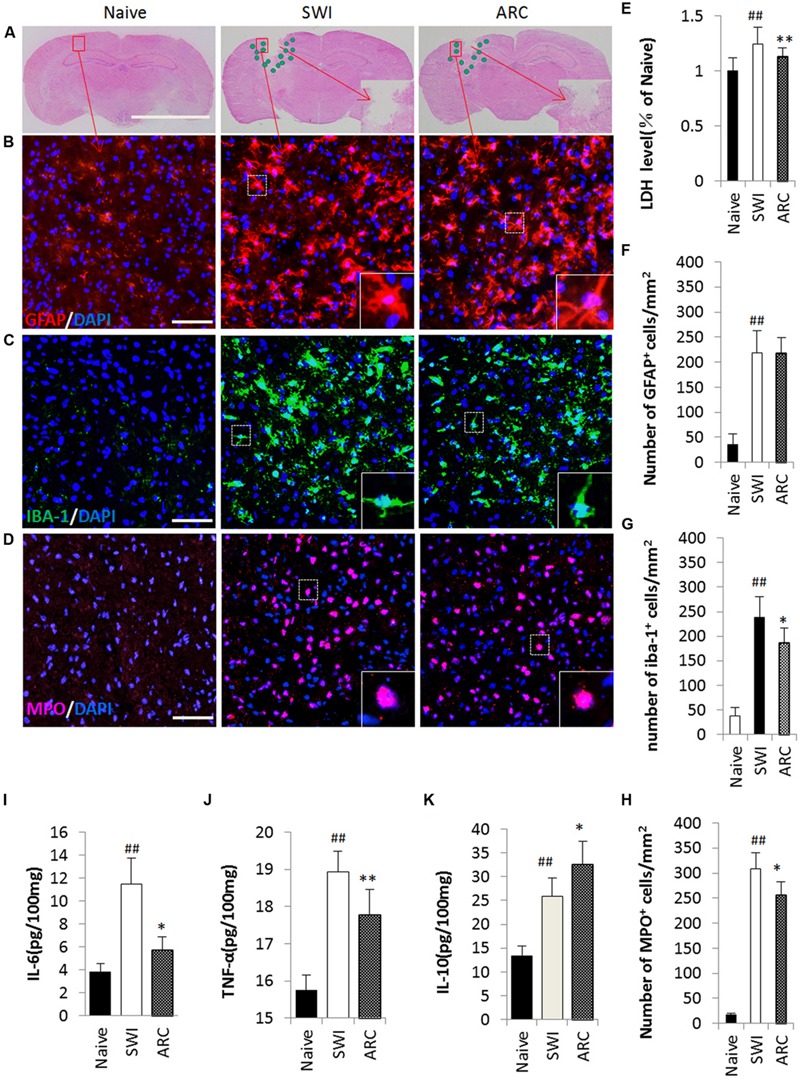
**Reduced inflammatory cell infiltration and pro-inflammatory cytokine levels as a result of ARC treatment. (A)** Illustration of the spatial distribution of inflammatory cells. The cells were distributed around the lesion at 3 dpi. **(B–D)** Images of GFAP+, Iba-1+, and MPO+ cells by immunohistochemistry in a single field (box) in the lesioned cortex. Macrophages/microglia (Iba-1+ green), neutrophils (MPO+, red) and astrocytes (GFAP+, red) with nuclear DAPI staining (blue). **(E)** LDH was measured to evaluate the extent of cell damage. **(F–H)** Quantification of the inflammatory cells among the total number of cells (DAPI+). All groups were examined in the regions immediately surrounding the lesion. Boxed areas appear at higher magnifications in the insets. Scale bar: 5 mm in **(A)**, 50 μm in **(B–D)**. **(I–K)** Levels of the proinflammatory cytokines IL-6, TNF-α, and IL-10 assessed by ELISA assay. The data are presented as the mean ± SD, *n* = 6 per group. ^##^*P* < 0.01 vs. naive control; ^∗^*P* < 0.05, ^∗∗^*P* < 0.01, vs. SWI control.

Lactate dehydrogenase (LDH) was used to evaluate the extent of cell damage. LDH is released during tissue damage and is a marker of common injuries. The brain tissue broke down and neural cells were damaged after SWI (Zhao et al., 2012), and then the damaged cells released inflammatory cytokines (Zhang et al., 2012; Gao et al., 2014). Pro-inflammatory cytokines can activate astrocytes, and in turn, activated astrocytes release pro-inflammatory cytokines such as IL-6 and TNF-α (Lau and Yu, 2001; Ziebell and Morganti-Kossmann, 2010). Macrophages and neutrophils act as the first and main forms of active immune defense, and microglia are a type of glial cells that are the resident macrophages in the brain. After TBI, neutrophils and microglia were activated, and this was the main feature of neuroinflammation (Wang et al., 2007; Clausen et al., 2009). Microglia can also release IL-6 and TNF-α. At 3 days after injury, a large number of inflammatory cytokines were released, and a large number of inflammatory cells were activated (**Figure 5A**). Thus, the time point 3 dpi was chosen to test the role of ARC in the inflammatory response (Xia et al., 2015). As shown in **Figures 5B–D**, the numbers of Iba-1+ macrophages/microglia and MPO+ neutrophils in the lesioned cortex significantly decreased in the ARC treatment group compare to the untreated group; however, the numbers of GFAP+ astrocytes did not change, and there was no significant difference in the results of the experiment. (GFAP+: 217.33 ± 35.30 vs. 219.311 ± 42.96 cells/mm2; Iba-1+: 115.33 ± 47.96 vs. 247.83 ± 52.29 cells/mm2; MPO+: 256.67 ± 61.99 vs. 309.06 ± 52.67 cells/mm2, *P* < 0.01) (**Figures 5F–H**). We also measured the levels of pro-inflammatory cytokines (IL-6 and TNF-α) and anti-inflammatory cytokines (IL-10) in cortical tissue homogenates by ELISA at 3 dpi. As shown in **Figures 5E,I,J,K**, ARC reduced the levels of LDH and pro-inflammatory cytokines TNF-α and IL-6 and increased the level of IL-10 in ARC-treated relative to untreated control animals (LDH: 1.13 ± 0.087 vs. 1.24 ± 0.15; TNF-α: 17.78 ± 0.55 vs. 18.93 ± 0.68 pg/100 mg; IL-6: 5.71 ± 0.041 vs. 11.47 ± 0.054 pg/100 mg; IL-10: 32.60 ± 0.068 vs. 25.85 ± 0.079 pg/100 mg; P < 0.01).

### ARC Treatment Reduces Apoptosis and Increases Neuronal Survival in the Injured Brain

**FIGURE 6:**
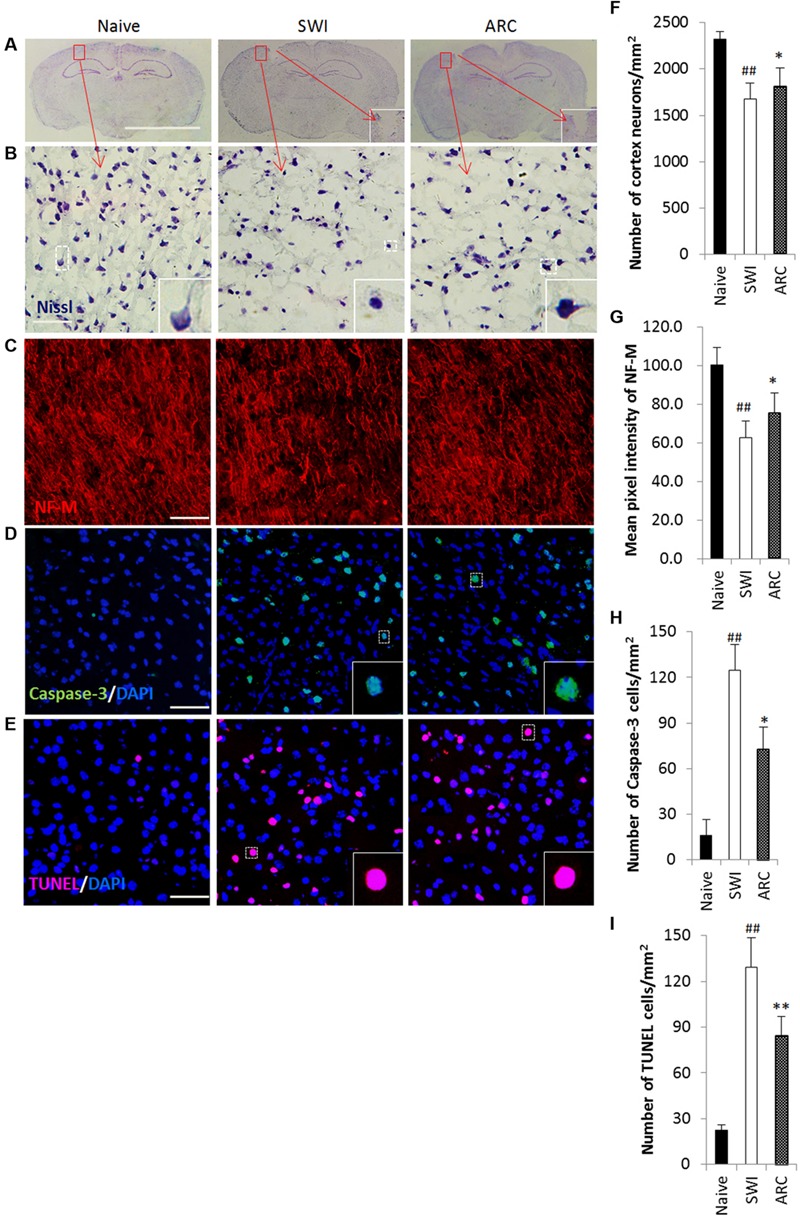
**Promoted neuronal survival and reduced apoptosis in lesioned brains of mice as a result of ARC treatment. (A–C)** Survival of neurons in the lesioned cortex of mice were stained with Nissl and anti-NF-M antibodies. **(D,E)** Apoptotic cells were detected by caspase-3 immunostaining and TUNEL assay on serial sections of the brain at 7 dpi. **(B,F)** Areas positive for Nissl antibody were revealed by Nssl staining. **(C,G)** Areas negative for NF-M were revealed by immunohistochemistry. **(D,H)** Apoptotic neurons were immunostained with anti-caspase-3^+^ antibodies. **(E,I)** Apoptotic cells were determined by TUNEL assay. The amount of NF-M expression around the lesions was quantified by measurement of pixel intensity of NF-M immunoreactivity by measurement of pixel intensity of NF-M immunoreactivity using Image J software. The amount of other positive expression around the lesions was quantified by measurement of number of positive cells using ImageJ software. Boxed areas appear at higher magnifications in the insets. Scale bar: 5 mm in A, 50 μm in **(B–E)**. Nuclei were stained with DAPI (blue). All groups were examined within the areas immediately surrounding the lesion. The data are presented as the mean ± SD, *n* = 6 per group. ^##^*P* < 0.01 vs. naive control; ^∗^*P* < 0.05, ^∗∗^*P* < 0.01, vs. SWI control.

The physical forces of TBI can shear axons (Raghupathi and Margulies, 2002) and break down neurons and its neighboring cells (**Figure 6A**), eventually resulting in axon loss and neuron and cell death (Clark et al., 1997). The most serious cell death occurred at 7 days after injury, and thus the time point 7 dpi was chosen to examine the role of ARC in the apoptotic response (Xia et al., 2015). To assess the effects of ARC treatment at the cellular level, brain sections were tested for Nissl staining (**Figures 6B,F**) and neurofilament medium polypeptide (NF-M) expression (**Figures 6C,G**) at 7 dpi, and apoptotic cells were detected by the TUNEL assay (**Figures 6E,I**) and by examining caspase-3 expression (**Figures 6D,H**). As Nissl stained images show in **Figure 6B**, the number of neuronal cells increased significantly in the ARC treatment group compared to untreated mice (Nissl+:113.17 ± 12.60 vs.105.34 ± 10.51) (**Figure 6F**). Axons decreased significantly in SWI control mice and resulted in lowered expression of NF-M. However, ARC treatment increased expression of NF-M in treated mice (**Figure 6C**). (fluorescence intensity: 75.55 ± 10.36 vs. 62.91 ± 8.32; *P* < 0.05) (**Figure 6G**). The number of TUNEL+ and caspase-3+ cells decreased significantly in the ARC-treated lesion area compared to untreated controls (TUNEL+: 84.25 ± 17.99 vs. 129.03 ± 23.73 cells/mm^2^; caspase-3+: 72.58 ± 14.54 vs. 124.81 ± 16.72 cells/mm^2^; *P* < 0.05) (**Figures 6D,E,H,I**). Thus, ARC promotes neuronal recovery by inhibiting apoptosis after SWI.

### ARC Treatment Suppresses the Inflammatory Response and Inhibits Apoptosis at the Gene Level

**FIGURE 7:**
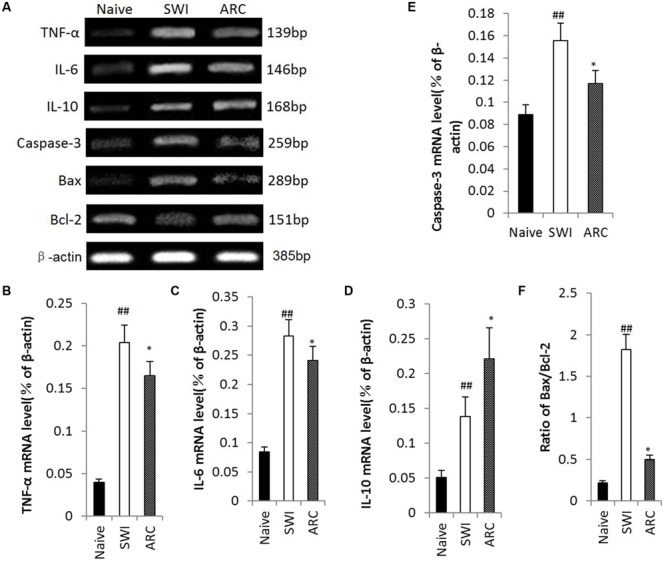
**Changes of gene expression in injured brain after ARC administration.** Total RNA was extracted from injured brain tissue at 3 and 7 dpi. mRNA expression of IL-6, IL-10 and TNF-α was examined at 3 dpi, and Bax, Bcl-2, and Caspase-3 were examined at 7 dpi. **(A)** mRNA expression revealed by RT-PCR. **(F)** Quantitative analysis of the ratio of Bax/Bcl-2. **(B–E)** Quantitative analysis of mRNA Levels of TNF-α, IL-6, IL-10, and Caspase-3. ARC administration up-regulated mRNA expression of IL-10, down-regulated mRNA expression of TNF-α, IL-6, and Caspase-3, and reduced the ratio of Bax to Bcl-2 significantly compared to the untreated control. The data are presented as the mean ± SD, *n* = 6 per group. ^##^*P* < 0.01 vs. naive control; ^∗^*P* < 0.05, ^∗∗^*P* < 0.01, vs. SWI control.

To clarify the potential mechanisms of anti-inflammatory and anti-apoptotic functions of ARC, we determined the levels of mRNA expression of pro-inflammatory cytokines (TNF-α, IL-6) and anti-inflammatory cytokine IL-10 and apoptotic factors caspase-3, Bax and Bcl-2 by RT-PCR. As shown in **Figure 7A**, SWI induced an increase in TNF-α, IL-6, IL-10, Bax, and Caspase-3 and a decrease in Bcl-2 transcription. ARC treatment resulted in downregulation of TNF-α, IL-6 and caspase-3, a reduction in the ratio of Bax to Bcl-2 and upregulation of IL-10 compared to the untreated SWI group (TNF-α: 0.165 ± 0.009 vs. 0.204 ± 0.015; IL-6: 0.241 ± 0.016 vs. 0.283 ± 0.018; IL-10: 0.221 ± 0.012 vs. 0.139 ± 0.012; caspase-3: 0.117 ± 0.010 vs. 0.012 ± 0.06; Bax to Bcl-2 ratio: 0.499 ± 0.083 vs. 1.823 ± 0.165; P < 0.01) (**Figures 7B–F**). Thus, ARC exerts anti-inflammatory effects via inhibition of TNF-α and IL-6 expression and promotion of IL-10 expression and confers anti-apoptotic effects by reducing the Bax to Bcl-2 ratio and downregulating caspase-3 expression.

## Discussion

After CED, the brain will be damaged by penetration as TBI. Following TBI, there are immediate primary effects and subacute secondary effects. Primary effects to the brain from TBI cause damage to neurons, glia, normal cells, and the cerebral vasculature. Secondary effects to the brain from TBI include inflammatory responses (Farook et al., 2013), cellular stress (Xia et al., 2015), apoptotic cascades (Clark et al., 1997), and loss of axons (Raghupathi and Margulies, 2002). Secondary effects are the main factors that affect the recovery of damage in later stages and are caused by primary effects. In this study, we mainly investigated the secondary injury treatment of ARC after CED.

In an animal model, we mimicked the procedure of CED by using a needle to create a SWI in the mouse brain and investigated whether ARC treatment could protect against secondary brain damage. In this study, ARC protected against BBB damage, reduced the number of microglia/macrophages in the brain cortex, decreased the number of peripheral infiltrating leukocytes at the lesion, reduced proinflammatory cytokine levels, increased anti-inflammatory cytokine levels, and inhibited apoptosis. These effects were exerted via upregulation of IL-10 and downregulation of IL-6, TNF-α, and caspase-3 expression and a reduction in the ratio of Bax to Bcl-2 at gene level. Thus, ARC can prevent secondary brain damage through anti-inflammatory as well as anti-apoptotic mechanisms.

Cerebral hemorrhage and cerebral edema occurred after CED due to the severely damaged BBB. Previous studies have indicated that the occurrence of cerebral hemorrhage is followed by BBB, which is a universal and crucial pathophysiological change (Kastrup et al., 2008). Intracerebral hemorrhage can lead to serious brain tissue damage, such as cerebral hematoma after disruption of the BBB. The level of severity of hematoma is closely related to the clinical prognosis of patients (Wang, 2010; Loftspring et al., 2011; Moxon-Emre and Schlichter, 2011). In this study, we investigated the brain water content, the BBB permeability and the brain hematoma volume as a result of ARC treatment at 3 dpi. The results showed that ARC treatment protected against BBB damage by reducing the brain water content, the BBB permeability and the volume of brain hematomas. The BBB will open again at 3 dpi due to the inflammatory response (Başkaya et al., 1997). In previous studies (Zhao et al., 2009; Kou et al., 2011) and this study, results indicate that ARC confers anti-inflammatory effects, which is the potential mechanism of BBB protection.

After TBI, the damaged cells released LDH and inflammatory cytokines that can cause neuroinflammation. Neuroinflammation is characterized by macrophage and neutrophil infiltration and microglia activation after brain injury (Wang et al., 2007; Clausen et al., 2009). The number of neuroinflammatory cells can predict the extent of brain recovery. However, the term neuroinflammation generally refers to more chronic, sustained injury while the responses of microglial cells contribute to and expand the neurodestructive effects, worsening the disease process (Streit, 2006). Microglia can activate the proinflammatory cytokines IL-1α, IL-1β, and TNF-α in the CNS (Wood, 2003). Proinflammatory cytokines such as TNF-α and IL-6 are mainly produced by microglia, which in turn activate glia, further stimulating cytokine production and astrogliosis (Lau and Yu, 2001; Ziebell and Morganti-Kossmann, 2010; Xia et al., 2012). In a previous report, ARC had significantly decreased MPO and EPO activities in injury tissues (Kang et al., 2008). Thus, in previous studies and in this study, results suggest that ARC suppresses trauma-induced inflammation by inhibiting microglia activation and neutrophil infiltration as well as the release of proinflammatory cytokines that can cause secondary damage to the brain after injury.

The expression of inflammatory cytokines TNF-α, IL-6, and IL-10 was upregulated after injury. TNF-α and IL-6 are proinflammatory cytokines (Wilson et al., 2005), and IL-10 is anti-inflammatory cytokine. Transgenic mice overexpressing IL-6 show enhanced neuroinflammation and brain damage (Campbell et al., 1993). TNF-α is a key cytokine in the inflammatory response caused by tissue destruction, bacterial infection and tumor cells (Aggarwal et al., 1985), and it can induce the occurrence of and worsen inflammation (Chen et al., 2006). The most important function of IL-10 is to limit and eventually terminate the inflammatory response (Moore et al., 2001). *In vitro*, IL-10 can significantly decrease the secretion of proinflammatory cytokines, such as TNF-α (Schmit et al., 2001). The data from Cuzzocrea et al. indicate that the reduction of TNF-α and IL-6 results in a decrease of secondary tissue injury (Genovese et al., 2008). In a previous study, ARC potently inhibited nitric oxide (NO), TNF-α and IL-6, but not cyclooxygenase (COX)-2 expression and COX-2 activity, possibly constituting the anti-inflammatory mechanism of ARC (Zhao et al., 2009). The data from Kou et al. (2011) shows that ARC exerts its anti-inflammatory effect by inhibiting reactive oxygen species (ROS)-dependent signal transducer and activator of transcription (STAT) signaling through its antioxidant activity. ARC also significantly reduced the phosphorylation of STAT1, STAT3, and JAK2 in LPS-stimulated RAW264.7 cells (Kou et al., 2011). ARC not only inhibited LPS-increased IL-6 and TNF-α expression in LPS-stimulated peritoneal macrophages but also increased LPS-reduced IL-10 and CD 204 expression (Hyam et al., 2013). Hence, excessive IL-6 and TNF-α-mediated inflammation is likely involved in the unfavorable outcomes associated with SWI. Based on the above data and these studies, ARC treatment partly blocks the increase in IL-6 and TNF-α expression and promotes the increase in IL-10 expression at the molecular and gene level as a result of brain injury, providing additional evidence that ARC suppresses neuroinflammation via downregulation of IL-6 and TNF- a and upregulation of IL-10.

The Nissl (Nissl, 1894) body is a type of alkaline substance that is widely distributed in various neurons, and it can be seen in the soma and dendrites of neurons though not in the axon or axon hillock (Thompson, 2000; Kühnel, 2003). Upon neuron damage, the number of Nissl bodies can be reduced or even disappear, thus the degree of damage of neurons can be observed by observation of the Nissl body (Zhao et al., 2014; Zhai et al., 2015). NFs are the most abundant cytoskeletal proteins in large myelinated axons (Hozumi et al., 1990; Perrot et al., 2008); specifically, NF-M is important for the stabilization of mature axons (Wood, 2003). NF-M expression is decreased in injured animals, indicating axonal loss. Neuron structure (perikaryon, axon, dendrite) will be destroyed after CED, leading to secondary injury. Secondary pathophysiological insults will cause delayed neuronal death in surrounding or distant regions (Stoica and Faden, 2010). We observed the number of Nissl staining neurons and NF-M expression around the cavity in injured animals, results show that ARC treatment increases the number of neurons and NF-M expression compared to untreated animals, providing structural evidence for the effects of ARC in promoting neuronal restoration and inhibiting apoptosis in the injured brain.

Caspase-3 is a caspase protein that is encoded by the *CASP3* gene. Caspase-3 is activated in the apoptotic cell both by the extrinsic (death ligand) and the intrinsic (mitochondrial) pathways (Salvesen, 2002; Ghavami et al., 2009), and caspase-3 activity induces apoptosis and will kill cells indiscriminately (Li et al., 1997; Boatright and Salvesen, 2003). The Bcl-2 family includes genes encoding the anti-apoptotic protein Bcl-2 and the pro-apoptotic protein Bax (Youle and Strasser, 2008; Kotipatruni et al., 2011). Bax overexpression stimulates apoptosis and suppresses the recovery of injury (Gross et al., 1998), while the overexpression of Bcl-2 inhibits neuronal apoptosis and promotes the recovery of neurological function (Allsopp et al., 1993; Fan et al., 2010). Bax upregulation and Bcl-2 downregulation increases the Bax to Bcl-2 ratio; this may be directly associated with cytochrome c release (Adams and Cory, 1998). In this study, ARC treatment reduced the Bax to Bcl-2 ratio and caspase-3 levels compared to untreated mice, providing insight into the mechanism underlying the anti-apoptotic effects of ARC.

Apoptosis is the process of programmed cell death (PCD) (Green, 2011). A form of traumatic cell death that results from acute cellular injury, apoptosis is a highly regulated and controlled process that confers advantages during an organism’s lifecycle. However, excessive apoptosis causes atrophy. Among the induction of apoptosis, inflammation plays an important role (Saal, 1995; Lucas et al., 2006). TNF-α is a cytokine mainly produced by activated macrophages and is the major extrinsic mediator of apoptosis (Harper et al., 2003; Yan et al., 2013). IL-6 is a pro-inflammatory cytokine that can also induce cell death (Yao et al., 2015), While IL-10 is an anti-inflammatory cytokine, it can also inhibit cellular apoptosis (Banz et al., 2002). In previous reports, ARC was shown to confer neuroprotective effects through anti-inflammatory and anti-apoptotic effects (Swarup et al., 2008). In the present study, we investigated the number of dead cells using a TUNEL assay and capsase-3 expression around the cavity in injured animals. The results show that ARC treatment decreases the number of TUNEL+ and caspase-3+ expressing cells compared to untreated animals, providing anti-inflammatory and anti-apoptotic evidence for the effects of ARC in promoting neuronal protection in the injured brain. Thus, we conclude that the underling mechanism of apoptosis inhibition of ARC occurs through an anti-inflammatory process.

## Conclusion

Arctigenin treatment protects against BBB damage and secondary brain injury via anti-inflammatory and anti-apoptotic mechanisms in an experimental cortical SWI model. This study demonstrates that ARC confers neuroprotection and improves long-term neurological outcomes after CED.

## Author Contributions

JY, TK, and JS designed and performed the experiments, drafted and revised the manuscript, and prepared the final version of the manuscript. NL, YX, YJ, JS, LK, YY, YJ, YY, SL, ZT, TK, and SZ performed the experiments and analyzed and interpreted the data. All authors read and approved the version submitted for publication.

## Conflict of Interest Statement

The authors declare that the research was conducted in the absence of any commercial or financial relationships that could be construed as a potential conflict of interest.
